# Genotyping Analyses of Tuberculosis Cases in U.S.- and Foreign-Born Massachusetts Residents

**DOI:** 10.3201/eid0811.020370

**Published:** 2002-11

**Authors:** Sharon Sharnprapai, Ann C. Miller, Robert Suruki, Edward Corkren, Sue Etkind, Jeffrey Driscoll, Michael McGarry, Edward Nardell

**Affiliations:** * Massachusetts Department of Public Health, Boston, Massachusetts, USA; † New York State Department of Health, Wadsworth Center, Albany, New York, USA; ‡ Harvard Medical School, Boston, Massachusetts, USA

**Keywords:** tuberculosis, epidemiology, molecular, DNA fingerprinting, cluster analysis, emigrant, immigrant

## Abstract

We used molecular genotyping to further understand the epidemiology and transmission patterns of tuberculosis (TB) in Massachusetts. The study population included 983 TB patients whose cases were verified by the Massachusetts Department of Public Health between July 1, 1996, and December 31, 2000, and for whom genotyping results and information on country of origin were available. Two hundred seventy-two (28%) of TB patients were in genetic clusters, and isolates from U.S-born were twice as likely to cluster as those of foreign-born (odds ratio [OR] 2.29, 95% confidence interval [CI] 1.69, 3.12). Our results suggest that restriction fragment length polymorphism analysis has limited capacity to differentiate TB strains when the isolate contains six or fewer copies of IS*6110*, even with spoligotyping. Clusters of TB patients with more than six copies of IS*6110* were more likely to have epidemiologic connections than were clusters of TB patients with isolates with few copies of IS*6110* (OR 8.01, 95%; CI 3.45,18.93).

The incidence of tuberculosis (TB) in the United States is closely linked to the global TB epidemic (1). In 2000, 46% of all reported TB cases in the United States occurred among persons not born there (foreign-born), and 20 states reported that >50% of TB cases occurred among the foreign-born (2). In Massachusetts, 202 (71%) of 285 cases reported were among foreign-born persons (from 41 different countries). Being born outside the United States is the primary risk factor for being reported with TB in Massachusetts (3).

The distribution of places of birth among TB patients reported in Massachusetts has changed greatly over the past 3 decades, reflecting changes in populations immigrating to Massachusetts. As late as 1970, 80% of foreign immigrants in Massachusetts were from Europe or Canada; only 5% of the immigrants were from Asia, and less than 3% were from Central and South America combined and Africa (4). Since 1970, the proportion of immigrants to Massachusetts from Europe has declined, and the proportion of those from Asia, the Caribbean Islands, Africa, and South and Central America has risen. Immigrants from Asia increased sharply, from 3% to 16%. Between 1996 and 2000, the proportion of foreign-born TB patients reported in Massachusetts rose from 61% to72%. This increase was seen primarily among Asians, Africans, and immigrants from Central and South America.

Understanding the factors that contribute to the incidence of TB is critical for TB elimination. Molecular fingerprinting data can be used to further an understanding of the epidemiology and transmission patterns of TB. In this article, we describe the epidemiology of TB patients in Massachusetts and results of using genotyping to evaluate the extent to which genetic clustering of *Mycobacterium* *tuberculosis* differs between foreign-born and U.S.-born TB patients.

## Methods

In 1996, the Massachusetts Department of Public Health, Division of Tuberculosis Prevention and Control (TB Division) became part of the Centers for Disease Control and Prevention (CDC)’s National Tuberculosis Genotyping and Surveillance Network. The TB Division attempted to locate and submit at least one isolate for every culture-confirmed TB case-patient reported from July 1, 1996, through December 31, 2000, to the Northeast Regional Genotyping Laboratory, New York State Department of Health, Wadsworth Center, Albany, New York. DNA genotyping by using IS*6110* restriction fragment length polymorphism (RFLP) and the spoligotyping technique (spacer oligotyping) was performed by the Wadsworth Center. RFLP analysis was performed by using the standard method (5,6) with the molecular weight standards provided by CDC. Spoligotyping was performed with a commercially available kit, in accordance with the manufacturer’s instructions (Isogen Bioscience BV, Maarseen, the Netherlands).

### Specimen Collection for DNA Fingerprinting Analysis

The following procedures were used to identify patients with positive *Mycobacterium tuberculosis* cultures and obtain isolates for RFLP analysis. In 1996, a survey of hospitals and private physicians was conducted to ascertain where specimens were being sent for mycobacterial culture. This survey allowed the TB Division to determine which laboratories inside and outside of the state were processing clinical specimens for Massachusetts residents. In addition, a letter was sent to directors of all laboratories in Massachusetts that are licensed under the Clinical Laboratory Improvement Act (CLIA) to perform mycobacteriology services and to other laboratories that were identified through the survey, asking for their cooperation with the TB genotyping network project. Most (71%) hospitals and physicians sent specimens to the Massachusetts State Laboratory Institute (MSLI) for culture identification, susceptibility testing, or both. The TB Division and the Mycobacteriology Laboratory,MSLI, share a joint database where all bacteriology reports, including drug susceptibility information, are automatically linked to suspected and confirmed cases of TB. For *M. tuberculosis* specimens that were processed elsewhere, the epidemiologists on the TB genotyping network project identified laboratories by attending routine TB case and cohort reviews conducted monthly by the state TB nurses and the Boston Public Health Commission TB Program. Laboratories were then contacted and arrangements were made for shipment of specimens to the MSLI and the Wadsworth Center.

### Cluster Investigation

RFLP analysis by using IS*6110* is a powerful tool for discerning one strain of *M*. *tuberculosis* from another when there are many copies of IS*6110*. However, for strains of *M. tuberculosis* with low copy numbers of IS*6110*, RFLP analysis has less discriminating power, and therefore a secondary typing method is used to help differentiate strains (7,8). For the TB genotyping network project, isolates were considered to be clonally related (i.e., were the same strain of TB) if they had identical IS6*110* patterns containing seven or more bands or they had identical IS6*110* patterns containing six or fewer bands with identical spoligotyping. A cluster was defined as containing two or more patients with clonally related TB strains.

In 1998, CDC funded the Cluster Investigation Study to evaluate epidemiologic links between clustered cases in a more formal manner. Cluster investigations consisted of standardized medical record reviews wherever a patient was seen for TB, and standardized interviews with the patient (or a proxy) if the patient was unable to participate. All patients were eligible for interview, unless strong epidemiologic links were found between all members of the cluster. In that situation, interviews were considered unnecessary. Written informed consent was obtained from all subjects, and interpreters were used as needed. Information collected through medical record reviews and patient interviews included the estimated period of infectivity, demographics, employment history, and social connections and activities during the 2 years before diagnosis. Each patient in a genetic cluster was examined to determine the following: 1) the period of infectivity (by reviewing date of diagnosis, disease type, smear status, chest radiology results, and date treatment started), 2) name of contacts identified, and 3) how and where the patient spent his or her time during the period of infectivity. If a patient identified another patient in the same cluster, or if patients were found to be in the same place at the same time when one was infectious, the likelihood of transmission was classified as “definite.” Transmission was “possible” if patients were thought to be at the same place, at the same time up to 2 years before diagnosis, or if patients identified the same contact as being the source of TB. A final category, “unlikely,” was designated when no common place or other epidemiologic connection was identified or when patients had arrived so recently in the country that transmission was unlikely to have occurred. Further details about the formal cluster investigation study are provided elsewhere (9). Data were analyzed by using Epi Info version 6.03 (10). The study was reviewed and approved by the, Human Research Review Committee, Massachusetts Department of Public Health.

## Results

### Epidemiology of TB in Massachusetts and Genotypes

From July 1, 1996, to December 31, 2000, a total of 1,281 cases were reported and verified as TB by the TB Division, of which 1,032 (81%) were confirmed with positive culture for *M. tuberculosis*. Genotype results were obtained for 984 (95%) of the culture confirmed cases. For the remaining 48 cases, genotype results were not obtained for a variety of reasons, including inability to obtain *M. tuberculosis* isolates from private laboratories and too little growth on culture. Of the 984 TB patients for whom DNA fingerprinting results were obtained, epidemiologic analyses were conducted for 983 patients whose country of origin was known. The greatest risk for developing TB in Massachusetts was being born outside the United States.

Six hundred eighty four (70%) of the TB patients were foreign-born (from 78 different countries). Most (295; 43%) foreign-born patients were from Asia, followed by the Caribbean region (118;17%) and Africa (116;17%). Countries with the highest number of cases included: Vietnam: 87 cases (13%); Haiti, 83 (12%); China, 59 (9%), India, 54 (8%); Cambodia, 31 (5%), and the Dominican Republic, 30 (4%). Analyses of intervals between arrival into the United States and diagnosis of TB indicated that 176 (26%) patients were diagnosed with TB within 1 year of arrival and 353 (52%) were diagnosed with TB within 5 years of arrival (Table 1).

**Table 1:** Tuberculosis (TB) cases in foreign-born persons by number of years in the United States, by geographic region

Time from arrival in the United States to TB diagnosis	Asia (%)	Caribbean (%)	Africa (%)	Europe (%)	South America (%)	Central America (%)	Former Soviet Union (%)	Other^a^(%)	Total (%)
<1 year	64 (22)	24 (21)	47 (41)	10 (29)	16 (34)	9 (22)	6 (27)	0	176 (26)
1–5 years	62 (21)	25 (21)	51 (44)	2 (6)	17 (36)	15 (37)	5 (23)	0	177 (26)
6–10 years	66 (22)	18 (15)	14 (12)	2 (6)	7 (15)	10 (24)	5 (23)	1 (12)	123 (18)
> 10 years	103(35)	51 (43)	4 (3)	23 (62)	7 (15)	7 (17)	6 (27)	7 (88)	208 (30)
Total (n=683)	295 (43)	118 (17)	116 (17)	37 (5)	47 (7)	41 (6)	22 (3)	8 (1)	684 (100)

^a^Other, 7 patients from Canada and 1 patient from Australia.

Foreign-born patients were likely to be younger than U.S.-born TB patients (Table 2). Three hundred twenty-seven (48%) of the foreign-born patients were ages 25–44, as compared to 75 (25%) of U.S.-born patients; 103 (15%) of foreign-born patients were >65 years, as compared with 108 (36%) of U.S.-born patients. Foreign-born patients were also more likely to have extrapulmonary disease: 232 (34%) of foreign-born patients had extrapulmonary TB compared with 61 (20%) of U.S.-born patients. TB patients born in the United States were more likely to have been homeless within the year before diagnosis, and drug use and excessive alcohol use were higher among U.S.-born patients than among foreign-born TB patients. Definition of drug use (injecting drug use and noninjecting drug use), homelessness, and excessive alcohol use are based on CDC criteria as contained in the instruction for the completion of the CDC TB cases reporting forms (11).

**Table 2:** Demographic characteristics of U.S.-born and foreign-born tuberculosis (TB) case-patients^a^

Demographics	Foreign-born (%) n=684	U.S.-born (%) n=299	Odds ratio and 95% confidence interval (CI)	p value
Sex				
Male	380 (56)	183 (61)	0.80 (95% CI 0.60, 1.06)	p=0.11
Female	304 (44)	117 (39)		
Age group^b^				
<1–24	106 (16)	23 (8)	1.0	
25–44	327 (48)	75 (25)	1.06 (95% CI 0.61,1.83)	p=0.83
45–64	148 (22)	93 (31)	2.90 (95% CI 1.67,5.04)	p<0.01
>65	103 (15)	108 (36)	4.83 (95% CI 2.77,8.47)	p<0.01
Site of disease^b^				
Pulmonary	452 (66)	238 (80)	0.50 (95 %CI 0.36,0.70)	p<0.01
Extrapulmonary	232 (34)	61 (20)		
HIV status				
Positive	59 (9)	31 (10)	0.75 (95% CI 0.44,1.30)	p= 0.28
Negative	177 (26)	70 (23)		
Unknown	449 (66)	198 (66)		
Homeless in past year^b^				
Yes	16 (2)	38 (13)	0.16 (95% CI 0.09,0.31)	p<0.01
No	666 (97)	258 (86		
Unknown	2 (<1)	3 (1)		
Drug use in past				
Year^b,c^				
Yes	6 (<1)	27 (9)	0.08 (95% 0.03,0.21)	p<0.01
No	611 (89)	221 (74)		
Unknown	67 (10)	51 (17)		
Excessive alcohol use in past year^b^				
Yes	34 (5)	74 (25)	0.14 (95% CI 0.09,0.22)	p<0.01
No	577 (84)	176 (59)		
Unknown	73 (11)	49 (16)		

^a^Definitions of homeless, dug use and alcohol use are based on criteria established by the Centers for Disease Control and Prevention. ^b^ Significant difference observed between U.S.-born and foreign-born at p<0.01. ^c^Includes both injecting and noninjecting drug users.

### Distribution of Genotypes

Analyses of RFLP distribution indicated that 208 (21%)of 983 isolates contained six or fewer copies of IS*6110*. Sixty-seven (22%) of the isolates from 299 U.S.-born TB patients contained few copies of IS*6110*, as did 141 (21%) of the 684 isolates from foreign-born TB patients. However, isolates from foreign-born patients differed substantially by geographic region and country of birth (Table 3).One hundred one (34%) of isolates from Asian patients contained few copies of IS*6110* compared with 4% of isolates from persons born in South America. In addition, 42 (48%) of isolates from Vietnam contained few copies of IS*6110* compared with 7 (12%) from China*.*

**Table 3:** *Mycobacterium tuberculosis* IS*6110* copy numbers in genotypes by geographic region

Geographic region	No. of isolates in foreign-born (%) (N=684)	Containing > 6 copies of IS*6110* (%)	Containing ≤6 copies of IS*6110* (%)
Asia	295	194 (66)	101 (34)
China	59	52 (88)	7 (12)
India	54	34 (63)	20 (37)
Vietnam	87	45 (52)	42 (48)
Other	95	63 (66)	32 (34)
Caribbean	118	111 (94)	7 (6)
Dominican Rep	30	30 (100)	0
Haiti	83	76 (92)	7 (8)
Other	5	5 (100)	0
Africa	116	97 (84)	19 (16)
Europe	59	55 (93)	4 (7)
South America	47	45 (96)	2 (4)
Central America	41	36 (88)	5 (12)
Other ^a^	8	5 (57)	*3* (43)

^a^Other, 7 patients from Canada and 1 patient from Australia.

### Genetic Clustering of TB Cases by Genotyping

Of isolates from 983 TB patients, 711 (72%) had DNA fingerprints unique among Massachusetts isolates. The remaining 272 (27.7%) were in 82 genetic clusters. However, 171 (22%) of the 775 isolates containing more than six copies of IS6*110* were in genetic clusters as compared to 100 (48%) of the 208 isolates containing few copies of IS*6110*. Of the 208 isolates, 158 (76%) clustered by IS*6110* alone; 100 (48%) of the isolates remained clustered even with the addition of spoligotyping data to further differentiate the TB strain. The genetic clusters were relatively small in size; 52 (63%) of 82 clusters contained only 2 people, 25 clusters (30%) contained 3–5 people, and the largest cluster contained 16 people. Among the 299 U.S.-born TB patients, 119 (40%) patients had isolates in genetic clusters; 180 (60%) of those had isolates with a unique fingerprint. These figures compare with 153 (22%) of the 684 foreign-born TB patients who had isolates in genetic clusters and 531 (78%) who had unique fingerprints. U.S.-born TB patients were more likely to cluster than foreign-born TB patients (odds ratio [OR] 2.29, 95% confidence interval [CI] 1.69, 3.12). Foreign-born patients who had lived longer in the United States were more likely to have isolates that clustered than were recent arrivals (chi square for trend 6.31, p<0.05). Overall, 29 (16%) of those diagnosed with TB within 1 year of arrival had isolates that clustered with others as compared to 38 (22 %) among those diagnosed from 1 to 5 years of arrival and 26% among those diagnosed >5 years after arrival (Table 4). Stratified analyses by age group (<25, 25–44, 45–64, >65) indicated that clustering was associated with increased time spent in the United States for all age groups; however, the association was strongest among those 25–44 years of age (p<0.05).

**Table 4:** Molecular clustering of tuberculosis (TB) cases among foreign-born persons by time to TB diagnosis after arrival in the United States

Time to TB diagnosis	Cluster (%)	Nonclustered (%)	Odds ratio and 95% confidence interval (CI)	Chi square for trend ^a^
<1 year of arrival	29 (16)	148 (84)	1.0	6.31 p =.012
1–5 years of arrival	38 (22)	139 (78)	1.40 (95% CI 0.79,2.47)	
>5 years of arrival	86 (26)	244 (74)	1.80 (95% CI 1.10, 2.95)	
Total	153 (22)	531 (78)		


^a^Statistically significant trend for overall link based on country of origin was observed at p<0.05

### Likelihood of Epidemiologic Link among Clustered TB Cases

Although the TB genotyping network was started in 1996, cluster investigation did not formally begin until 1998. Therefore, of the 272 patients found in 82 clusters overall, only 161 patients in 52 clusters were investigated for epidemiologic connections as part of the formal Cluster Investigation Study. Information regarding epidemiologic connections was obtained for 152 (94%) of 161 patients. Epidemiologic connections were established for 68 (45%) of the 152 clustered TB cases, but none were found for 84 (55%) of the clustered TB cases. Epidemiologic connections were more likely to be identified for clusters containing only U.S.-born TB patients than clusters containing some or all foreign-born TB patients (62% vs. 42% and 33%, respectively; chi square for trend, p<0.05). In addition, clustered TB patients with many copies of IS*6110* were more likely to have epidemiologic connections than clusters with few copies of IS*6110* (OR 8.01; 95% CI 3.45,18.93). Of the 90 clustered TB isolates with many copies of IS*6110*, 57 (63%) had epidemiologic connections identified, compared with the 11 (18%) epidemiologic connections that were identified among the 62 clustered TB case-patients with few copies of IS*6110*. Among the U.S.-born patients, 26 (79%) of the 33 patients with many copies of IS*6110* had definite or possible epidemiologic connections, whereas none of the 9 patients with few copies of IS*6110* had connections (Table 5).

**Table 5:** Epidemiologic connection among clustered tuberculosis (TB) cases in Cluster Investigation Study, 1998–2000

Cluster contains	Overall (%)^a^			Many (>6 copies of IS*6110*) (%)			Few (≤6 copies of IS*6110*) (%)		
	No. Cases	Definite/possible	Unlikely	No. Cases	Definite/possible	Unlikely	No. Cases	Definite/possible	Unlikely
U.S.-born only	42	26 (62)	16 (38)	33	26(79)	7(21)	9	0	9 (100)
U.S.-born and foreign-born	67	28 (42)	39 (58)	30	19 (63)	11 (37)	37	9 (24)	28 (76)
Foreign-born only	43	14 (33)	29 (67)	27	12 (44)	15 (56)	16	2 (13)	14 (87)
Total	152	68 (45)	84 (55)	90	57 (63)	33 (37)	62	11 (18)	51 (82)

^a^%, epidemiologic link.

Of the 152 clustered TB patients, 42 (28%) were in clusters containing only U.S.-born patients, 67 (44%) were in clusters with mixed U.S.-born and foreign-born patients, and 43 (28%) were in clusters containing only foreign-born patients. Analysis of the 67 TB patients in mixed clusters containing both U.S.-born and foreign-born persons indicate that 38 (57%) of the TB patients were foreign-born, and 29 (43%) were U.S.-born. Epidemiologic connections were established for 28 (42%) of the 67 TB patients in mixed clusters, and the 17 resulting relationships were analyzed to determine the direction of TB transmission between the cluster members. Results indicate that TB was transmitted from foreign-born to U.S.-born persons in 6 (35%) relationships, foreign-born to foreign-born persons in five (29%) relationships, U.S.-born to U.S.-born persons in three (18%) relationships and U.S.-born to foreign-born persons in three (18%) relationships. However, three of the six foreign-born to U.S.-born relationships involved children of foreign-born parents born in the United States. Epidemiologic relationships were established for 26 (62%) of the 42 TB patients in clusters containing only U.S.-born persons, resulting in 20 relationships. Of the 43 TB patients in clusters containing only foreign-born persons, epidemiologic connections were established for 14 patients (33%), resulting in eight relationships. Overall, of the 45 relationships established through the 68 clustered TB patients with epidemiologic connections, possible TB transmission between U.S.-born persons occurred in 23 (51%) relationships, from foreign-born to foreign-born persons in 13 (29%) relationships, from foreign-born to U.S.-born in 6 (13%) relationships and from U.S.-born to foreign-born in 3 (7%) relationships. In addition, of the 38 foreign-born TB patients in mixed U.S.-born and foreign-born clusters, 10 (26%) TB was diagnosed within 1 year of arrival, in 7 (18%), TB was diagnosed from 1–5 years of arrival, and among 21 (55%), TB was diagnosed > 5 years after the person arrived in the United States. However, TB patients in mixed clusters were no more likely than patients in clusters containing only foreign-born persons to be diagnosed with TB within 1 year, from 1–5 years, or >5 years of arrival (chi square for trend 0.038, p=0.85).

## Discussion

The greatest risk of developing TB in Massachusetts is being foreign-born. This finding is consistent with the results found by Mitnick et al., indicating that the foreign-born were 7.5 times more likely to have TB than U.S.-born residents of this state (3). An analysis of time from arrival to TB diagnosis indicated that among 26%,TB was diagnosed within 1 year of arrival and among another 26%, it was diagnosed from 1 to 5 years of arrival. This increased risk soon after arrival is particularly true for persons arriving from Africa and South America, among whom TB was diagnosed within 1 year of their arrival for 41% and 35%, respectively,. In Massachusetts, the TB Division is notified of refugees and immigrants with a class A or B TB condition identified through the overseas screening process. Together with the Massachusetts Refugee and Immigrant Health Program, the TB Division works to ensure that those refugees and immigrants are evaluated for active TB soon after their arrival in the United States. However, most foreign-born persons moving into Massachusetts are not refugees or immigrants but students or tourists, and therefore the TB Division has little or no information that would allow targeted TB screening.

Only 28% of Massachusetts TB patients had *M. tuberculosis* isolates that were clonally related. Most TB cases were likely the result of reactivation of old infection or recent infection that occurred in the person’s country of origin, rather than new infection acquired in this state.. U.S.-born patients were twice as likely to cluster as foreign-born TB patients, suggesting that transmission may be occurring more in the U.S.-born population. U.S.-born TB patients were significantly more likely than foreign-born patients to have a communicable form of TB disease, which may be one more explanation for the increase in clustering among U.S.-born patients. TB transmission between foreign-born and U.S.-born cluster members was established in 9 (20%) of the clustered TB patients with epidemiologic connections; however, to fully examine the extent that U.S.-born and foreign-born TB patients transmit TB in Massachusetts requires a longitudinal investigation of contacts, which was beyond the scope of this investigation. In addition, among those not born in the United States, increased time spent in the United States and clustering appeared to be related. Thus, TB that developed soon after the arrival of the foreign-born appeared to have been acquired abroad, and more of the later onset cases in foreign-born persons appeared to be due to infection acquired in Massachusetts.

The comparison between genotype clustering and epidemiologic connection provides evidence that the ability of DNA fingerprints to differentiate TB strains is limited when there are few copies of IS*6110.* Only 37% of the isolates in clusters containing few copies of IS*6110* had their TB strain differentiated further by spoligotyping. Examination of clustered TB patients with no epidemiologic links indicated that two thirds had few copies of IS*6110.* Epidemiologic connections were more often discovered when the clusters involved U.S.-born TB patients. Despite the use of interpreters, we may have been less successful in obtaining epidemiologic relationship information from foreign-born patients than from U.S.-born patients because of language and cultural barriers. However, even in the clusters of the U.S.-born patients, in which language was not an issue, epidemiologic connections could not be found in clusters with few RFLP bands. This suggests that the use of RFLP analysis, even with spoligotyping, may not be powerful enough to identify true clustering among isolates with few copies of IS6*110*.

The drawbacks to the RFLP technique include the following: it is labor-intensive, requires culture growth, is difficult to reproduce, and can require laborious secondary typing techniques (7,8,12). Other genotyping techniques, such as mycobacterial interspersed repetitive units–variable number of tandem repeats, are being considered that may offer advantages, including rapid turnaround time for results, reproducibility, and high sensitivity and specificity for *M. tuberculosis*. However, those methods may have less discriminating power than RFLP (7,12). Analyses of distribution and clustering of RFLP patterns may provide information regarding the ability of RFLP and other possible DNA fingerprinting methods to differentiate TB strains within various communities. For example, our analysis suggests that the ability of DNA fingerprinting to differentiate TB strains in the Asian community may be limited because one third of the isolates contained few copies of IS*6110,* and the secondary fingerprinting technique had less discriminatory power (Table 3).

Some limitations of the study must be noted. First, in RFLP analysis, the usual turnaround time between specimen collection and availability of result is lengthy (7,8). In some years, our turnaround time averaged 8 months. This lag time hindered the program’s ability to locate clustered patients for interview and affected the patients’ ability to recall contacts, and thus could have contributed to the relatively low percentage of completed interviews (65%). Of 56 patients eligible for interviews, 41% were lost to follow-up or had moved out of state.

Other limitations include the lack of specificity to differentiate TB strains with few copies of IS6110 (7) and incomplete sampling (13). An overestimation of genetic clustering, particularly among isolates with few copies of IS*6110,* may have occurred. On the other hand, clustered TB patients may have been underestimated because possible clonal relationships of isolates from our study population may have existed with patients reported outside of Massachusetts or outside the study time frame. In addition, a certain number of isolates in every population are unable to be given RFLP types.

## Conclusions

Molecular fingerprint data were useful in describing the epidemiology of TB in Massachusetts. Using this information, the TB Division can estimate TB patients that resulted from transmission in this state and design appropriate interventions. However, the capacity of DNA fingerprinting data to differentiate TB stains may vary by community of interest, and RFLP analysis, even with secondary typing, may not identify true clusters when isolates have few copies of IS6*110.* This situation has implications for genotyping techniques that have less discriminatory power than RFLP analysis. DNA fingerprinting should therefore be used in conjunction with effective surveillance and appropriate epidemiologic investigation.
